# Serum biomarkers associated with baseline clinical severity in young steroid-naïve Duchenne muscular dystrophy boys

**DOI:** 10.1093/hmg/ddaa132

**Published:** 2020-06-27

**Authors:** Utkarsh J Dang, Michael Ziemba, Paula R Clemens, Yetrib Hathout, Laurie S Conklin, CINRG Vamorolone 002/003 Investigators, Eric P Hoffman

**Affiliations:** 1 Department of Health Outcomes and Administrative Sciences, School of Pharmacy and Pharmaceutical Sciences, Binghamton University—SUNY, Binghamton, NY 13902, USA; 2 Department of Biomedical Engineering, Watson School of Engineering, Binghamton University—SUNY, Binghamton, NY 13902, USA; 3 Department of Neurology, School of Medicine, University of Pittsburgh, Pittsburgh, PA 15213, USA; 4 Department of Veteran Affairs Medical Center, Pittsburgh, PA 15213, USA; 5 Department of Pharmaceutical Sciences, School of Pharmacy and Pharmaceutical Sciences, Binghamton University—SUNY, Binghamton, NY 13902, USA; 6 ReveraGen BioPharma, Rockville, MD 20850, USA

## Abstract

Duchenne muscular dystrophy (DMD) is caused by loss of dystrophin in muscle, and while all patients share the primary gene and biochemical defect, there is considerable patient–patient variability in clinical symptoms. We sought to develop multivariate models of serum protein biomarkers that explained observed variation, using functional outcome measures as proxies for severity. Serum samples from 39 steroid-naïve DMD boys 4 to <7 years enrolled into a clinical trial of vamorolone were studied (NCT02760264). Four assessments of gross motor function were carried out for each participant over a 6-week interval, and their mean was used as response for biomarker models. Weighted correlation network analysis was used for unsupervised clustering of 1305 proteins quantified using SOMAscan^**®**^ aptamer profiling to define highly representative and connected proteins. Multivariate models of biomarkers were obtained for time to stand performance (strength phenotype; 17 proteins) and 6 min walk performance (endurance phenotype; 17 proteins) including some shared proteins. Identified proteins were tested with associations of mRNA expression with histological severity of muscle from dystrophinopathy patients (*n* = 28) and normal controls (*n* = 6). Strong associations predictive of both clinical and histological severity were found for ERBB4 (reductions in both blood and muscle with increasing severity), SOD1 (reductions in muscle and increases in blood with increasing severity) and CNTF (decreased levels in blood and muscle with increasing severity). We show that performance of DMD boys was effectively modeled with serum proteins, proximal strength associated with growth and remodeling pathways and muscle endurance centered on TGFβ and fibrosis pathways in muscle.

## Introduction

Duchenne muscular dystrophy (DMD; OMIM #310200) is an X-linked genetic disorder that affects approximately 1 in 5000 male births and manifests in early childhood (2 to 6 years of age). The disorder is caused by loss-of-function mutations in the *DMD* gene that encodes for the dystrophin protein, and this loss of dystrophin in muscle tissues leads to progressive muscle weakness and wasting and early death (1,2). Current standard of care for DMD patients includes high-dose glucocorticoids (prednisone, deflazacort) that reduce muscle inflammation and delay loss of motor functions (3). There has been a recent acceleration in clinical research of possible pharmacological therapies for DMD, including studies aimed at restoring dystrophin in skeletal muscle, and disease-modifying agents targeting inflammation and other pathogenic aspects of the disease.

Patients with DMD all share loss of dystrophin protein in muscle from fetal life onwards. Despite this common biochemical event that initiates the disease process, there is marked variability in patient presentation and progression, and this complicates the interpretation of drug effectiveness in clinical trials. One approach to understanding and potentially controlling for clinical heterogeneity is to define clinical outcomes at a young age that are predictive of later losses of gross motor skills and quality of life changes. Using an analysis of 6 min walk data in 96 boys from a natural history study of Italian DMD patients (96% steroid-treated), the authors grouped patients into 4 subgroups (Classes I–IV) based on their disease progression, with loss of ambulation ranging from ~10 years of age (Class I) to ~20 years of age (Class IV) (4). A natural history study of 440 DMD patients followed up to 10 years showed that assessment of time to stand from supine (TTSTAND) at a young age was predictive of later loss of ambulation (3). These studies demonstrate the ability to use early outcomes as predictive of clinical variability, but do not address the biological causes of this variability.

One approach to defining possible biological causes of clinical variability is through definition of genetic modifiers in DMD—common polymorphisms in human populations that can be shown to modify DMD disease progression or response to glucocorticoid therapy. To date, DNA polymorphisms encoding two components of the TGFβ cell repair and fibrosis pathway (osteopontin [*SPP1* gene], latent TGFβ-binding protein 4 [*LTBP4* gene]), an inflammatory protein (CD40) and a loss-of-function polymorphism of α-actinin 3 (*ACTN3* gene) have been found to be associated with DMD severity and response to steroids (5–9). As with most genetic modifiers, these polymorphisms seem to explain a relatively small part of observed variability.

A second approach to define biological determinants of variable clinical phenotypes are via serum biomarkers (proteins, microRNAs). Serum biomarkers have been studied throughout DMD disease progression, and specific biomarkers associated with specific stages of the progressive disease (10,11). Glucocorticoid-responsive serum biomarkers have been defined in DMD and other disorders, and these same biomarkers have shown to be responsive to an experimental drug in clinical testing, vamorolone (9–11).

No studies to date have studied serum biomarkers associated with variable clinical severity within a specific age range. Given the progressive nature of DMD, we hypothesized that studies of biomarkers associated with severe or mild disease at a specific age should focus on younger DMD boys in a narrow age range and, further, that subjects studied were steroid-naïve—thus avoiding confounding variables of both age and drug treatment. We also hypothesized that such studies might be most accurately done in the context of a formal clinical trial of a new chemical entity, where standard operating procedures for both sample collection and training of clinical evaluators might lead to more robust findings.

Here, we report multivariate models of biomarker/phenotype associations in steroid-naïve patients with DMD, 4 to <7 years (*n* = 48). Studies were limited to baseline data of subjects enrolled in a clinical trial of vamorolone, a first-in-class partial agonist of the glucocorticoid receptor (12,13).

## Results

### Correlation of clinical outcomes at baseline

The VBP15–002 clinical trial (ClinicalTrials.gov NCT02760264) enrolled steroid-naïve DMD participants, 4 to <7 years (*n* = 48), at 11 expert sites in the Cooperative International Neuromuscular Research Group (CINRG; www.cinrgresearch.org) over a 14-month period (April 2016–June 2017). Clinical assessments included five motor function tests. Time to stand from supine (TTSTAND), time to run/walk 10 m (TTRW) and time to climb 4 stairs (TTCLIMB) were each expressed as a velocity. The 6 min walk test (6MWT) was expressed in meters walked, and the North Star Ambulatory Assessment (NSAA) was scored for 17 standard tests of function. For each motor outcome, four separate measures were done for each subject over a 6-week time frame (screening, baseline, Week 2, Week 4). For the 2-week period between baseline and Week 2, subjects were treated with daily doses of vamorolone in four dose groups (0.25, 0.75, 2.0, 6.0 mg/kg/day) (*n* = 12 per group). For the 2-week period between Week 2 and Week 4, subjects had no drug treatment (washout). There were no significant effects of drug on outcomes in the short 2-week treatment period. We thus considered the four assessments in 6 weeks as ‘repeated measures’ for each subject, and motor outcome values were expressed as the mean of the four repeated measures. This mitigates intra-subject variation and accuracy issues inherent with motor outcome testing. We also provide summaries of coefficients of variation (%CV) for each of the outcomes for 48 subjects (Table 1). The averaged %CV for TTSTAND velocities and 6MWT are 16.17 and 7.17%, respectively.

**Table 1:** Coefficient of variation was calculated for 48 DMD boys for the four repeated measurements of each motor outcome and velocity transformation taken over 6 weeks

	TTSTAND velocity	TTSTAND seconds	TTCLIMB velocity	TTCLIMB seconds	TTRW velocity	TTRW seconds	6MWT	NSAA
Mean	16.17%	16.42%	13.61%	14.31%	7.61%	7.68%	7.17%	7.76%
SD	8.44%	9.06%	6.57%	7.57%	3.75%	3.97%	4.29%	5.86%

The mean (mean %CV) and standard deviation of these are reported.

**Figure 1:**
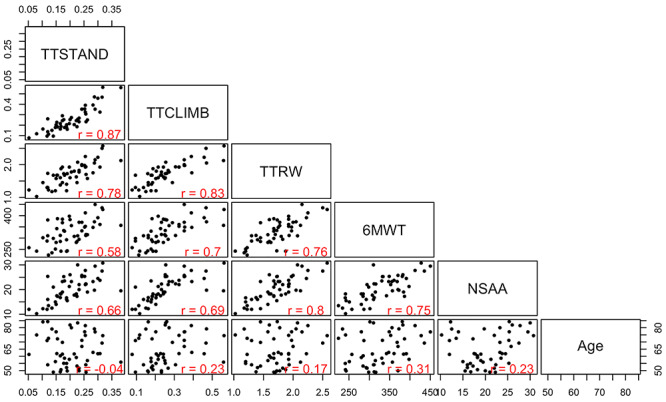
Scatterplot matrix of timed function tests, 6MWT, NSAA and age. Shown is mean of four measures over ~6-week time frame for 48 DMD subjects (4 to <7 years, steroid-naïve). The first three motor outcomes, TTSTAND, TTCLIMB and TTRW, are quantified in velocity units, 6MWT in meters, NSAA out of a maximum of 34 and age in months. Pearson correlations are also provided. The figure in the first column, second row, represents a scatterplot of time to stand (*x*-axis) versus time to climb (*y*-axis).

Correlations of each clinical outcomes were done with each other and with age for all 48 subjects (Fig. 1). In terms of correlations of clinical outcomes, TTSTAND and TTCLIMB were best correlated (*r* = 0.87); this likely reflects the shared proximal muscle strength required for these outcomes. Correlations for all other comparisons ranged from *r* = 0.58 to *r* = 0.83. There was no substantial correlation of age with outcomes; this was expected given that we studied a narrow age range (4 to <7 years) and that in this age range, DMD patients are typically stable in terms of disease progression (honeymoon period) (3). As DMD is a progressive disease, the lack of correlation with age in our data set suggests we are isolating intrinsic differences in inter-patient disease severity.

### Definition of clusters of serum protein biomarkers

Protein profiling of serum samples of DMD subjects enrolled in VBP15–002 (*n* = 39) using SOMAscan^®^ aptamer measures of 1305 serum proteins was previously described (12). Data were not available for nine subjects. SOMAscan^®^ analysis tested relative levels of all serum proteins tested using three dilutions of sera over a >10 000-fold dynamic range. In the previous report (12), analyses of SOMAscan^®^ data were restricted to testing drug responsiveness of 13 pre-specified pharmacodynamic biomarkers that had been identified as responsive to glucocorticoids (14). This is the first report of analysis of the complete 1305 serum protein data set, and here we restrict analyses to blood samples taken at baseline visits.

**Figure 2:**
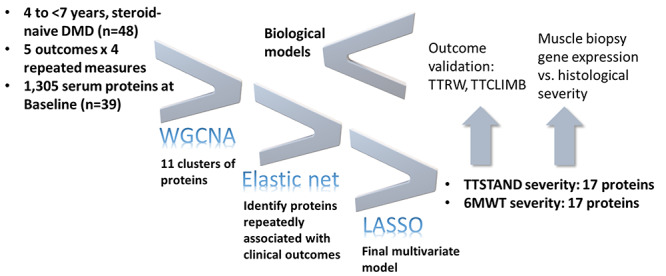
Overview of experimental design to define disease severity biomarkers in DMD. Sequential data reduction approaches were used for TTSTAND and 6MWT motor outcomes (WGCNA). The resulting regression model included 17 serum proteins associated with TTSTAND severity (proximal strength) and 17 proteins associated with 6MWT severity (endurance). The selected serum proteins were tested for gene expression associated with disease status and histopathological severity and were also validated in additional outcomes (TTRW and TTCLIMB from TTSTAND model). Finally, the resulting data were used to build hypothetical biological models for disease severity in DMD.

To identify sets of serum biomarkers associated with DMD subject clinical severity at baseline, we carried out a three-step approach, separately for two outcome measures (TTSTAND velocity reflective of proximal muscle strength; 6MWT meters reflective of muscle endurance). The first step accomplished data reduction using weighted gene correlation network analysis (WGCNA) (15) and summary measures of clusters (module eigenproteins; MEs). The second step used elastic net models (16) with repeated cross-validation to further reduce dimensionality to a few dozen proteins. The final step was application of least absolute shrinkage and selection operator (LASSO) (17) to arrive at 17 proteins each that were associated with clinical severity for TTSTAND and 17 associated with 6MWT (Fig. 2).

For the initial WGCNA/ME step, we analyzed relative trends of correlated serum proteins using a protein relative expression similarity network built with a weighted correlation network approach (15,18). A hierarchical clustering of proteins based on a transformation of pairwise correlations was used to identify protein clusters that were associated with clinically phenotypes. The initial clustering is conducted in a completely unsupervised manner (not connected to any outcomes or traits) at this stage of the analyses.

**Figure 3:**
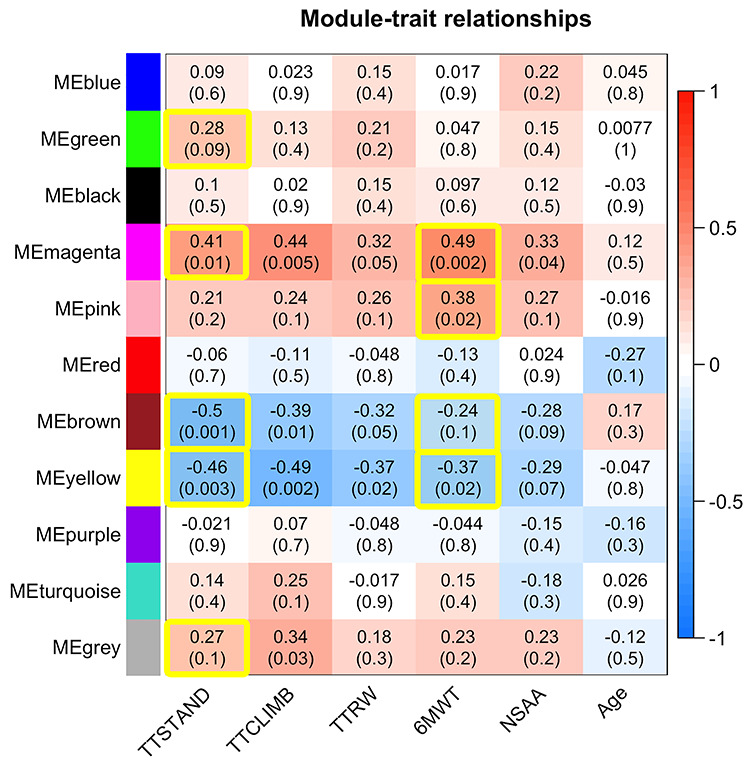
Screening step showing module trait relationships between motor outcomes and modules of serum proteins. Correlations of 11 MEs for clusters of proteins with clinical outcomes. Pearson correlations and associated *P*-values in parenthesis are provided. *P*-values were not adjusted for multiple testing in this screening step. The yellow-highlighted cells were those carried forward for statistical model building for TTSTAND and 6MWT.

Weighted correlation network analysis of the 1305 serum proteins in 39 subjects yielded 11 modules (clusters) of proteins, with membership ranging from 17 to 287 proteins, with high topological overlap (Fig. 3). Correlations of four timed function tests and NSAA with the summary expression (MEs) of these modules were computed. For example, in Figure 3, TTSTAND, the first column, has a correlation of −0.5 with the ME for the brown module (*P* = 0.001), −0.46 with yellow module (*P* = 0.002) and so on. This shows that the brown and yellow protein modules are negatively correlated with time to stand velocity at baseline, while the magenta module is positively correlated with time to stand velocity at baseline. This analysis was used only for filtering and dimension reduction; therefore, no correction for multiple testing was done. Consistency of module–trait relationships across different clinical endpoints was evident, consistent with the correlation matrix between different motor outcomes (see Fig. 1). Importantly, patient age was not highly statistically significantly associated with any cluster, likely due to the narrow age range studied (4 to <7 years). Note that the gray module consists of proteins that could not be clustered well in other modules.

We then moved to statistical model building focused on two clinical outcome measures that are most divergent, as they are thought to represent different phenotypic variables: TTSTAND (primary outcome in the vamorolone clinical trials; proximal muscle strength) and 6MWT (highly responsive to drug in the vamorolone clinical trials; muscle endurance). We set an arbitrary cutoff of *P* < 0.15 (for higher power screening because of low sample size) for retention of modules into statistical model building. The correlations for clusters carried forward into the analysis for TTSTAND and 6MTWT are enclosed in yellow in Figure 3.

### Feature selection and model building

TTSTAND was chosen for initial model building, as it was the primary outcome in the clinical trial; shorter TTSTAND in younger patients with DMD has been linked to delayed disease progression (e.g. loss of ambulation) and improved survival (3,13). Using proteins within the five selected modules for TTSTAND velocity (Fig. 3), data reduction using multivariate linear models was carried out. Elastic net methods were used to establish variable importance measures by indicating how often a serum protein was picked in 100 cross-validations with TTSTAND velocity measures. A threshold of >60/100 cross-validations led to the candidate proteins being used as explanatory variables in a final LASSO model; this selected 17 serum proteins in the multivariate linear model. Note that of the final 17 proteins, BAD was selected from all 100 cross-validations, with the others being EPHA3 (98), IGF-I sR (90), semaphorin-6A (90), MK01 (84), eIF-5A-1 (73), ERBB4 (72), NSF1C (72), PAFAH beta subunit (72), SNAA (72), SOD (72), DLRB1 (71), PLPP (71), RAN (71), PA2G4 (70), Rab GDP dissociation inhibitor beta (69) and IFN-g R1 (67). Estimated regularized coefficients for the LASSO model are provided in Supplementary Material, Table S1.

6MWT meters walked was chosen as a second clinical outcome for establishing a multivariate linear model. 6MWT is considered a clinical outcome reflective of muscle endurance (and not strength) and showed the most significant drug dose–response in the vamorolone clinical trial (13). The 6MWT has also been used as the primary outcome in multiple DMD clinical trials (19,20). Four modules (Fig. 3) were selected for modeling of 6MWT clinical associations, three of which overlapped with the modules selected for TTSTAND. For 6MWT, the following 17 proteins were selected where elastic net cross-validation analyses showed selection of each serum protein in >60/100 tests followed by a final LASSO model for the candidates. Serum proteins selected were angiopoietin-1 (98), CBG (98), CTAP-III (98), ERBB4 (98), TGF-b2 (98), 4EBP2 (97), AMNLS (97), EPHA3 (97), nidogen (97), SOD (95), PDGF-AA (93), ON (74), BCL6 (71), CNTF (71), ubiquitin+1 (71), MAPK5 (66) and PLPP (61). Four serum proteins were shared between the TTSTAND velocity model and the 6MWT model (SOD, PLPP, EPHA3, ERBB4). Estimated regularized coefficients for the LASSO model are provided in Supplementary Material, Table S2.

For squared correlations between observed average (of four measurements) outcome and multivariate model predicted values, we obtained 0.87 for TTSTAND and 0.89 for 6MWT. As a sort of a sensitivity analysis, we correlated the predictions for TTSTAND using the obtained final model (coefficients) with the four sets of observed outcomes. Using the four sets of outcomes, we obtained the following squared correlation values: 0.64, 0.65, 0.76 and 0.80. Similarly, doing this for 6MWT distances, we obtained the following squared correlation values: 0.68, 0.74, 0.79 and 0.82. That these squared correlations are lower than those obtained with the data the models were trained on is not surprising, and the model overall shows good performance (also reflecting reliability of the repeated measures).

### Patient-level data visualization

**Figure 4:**
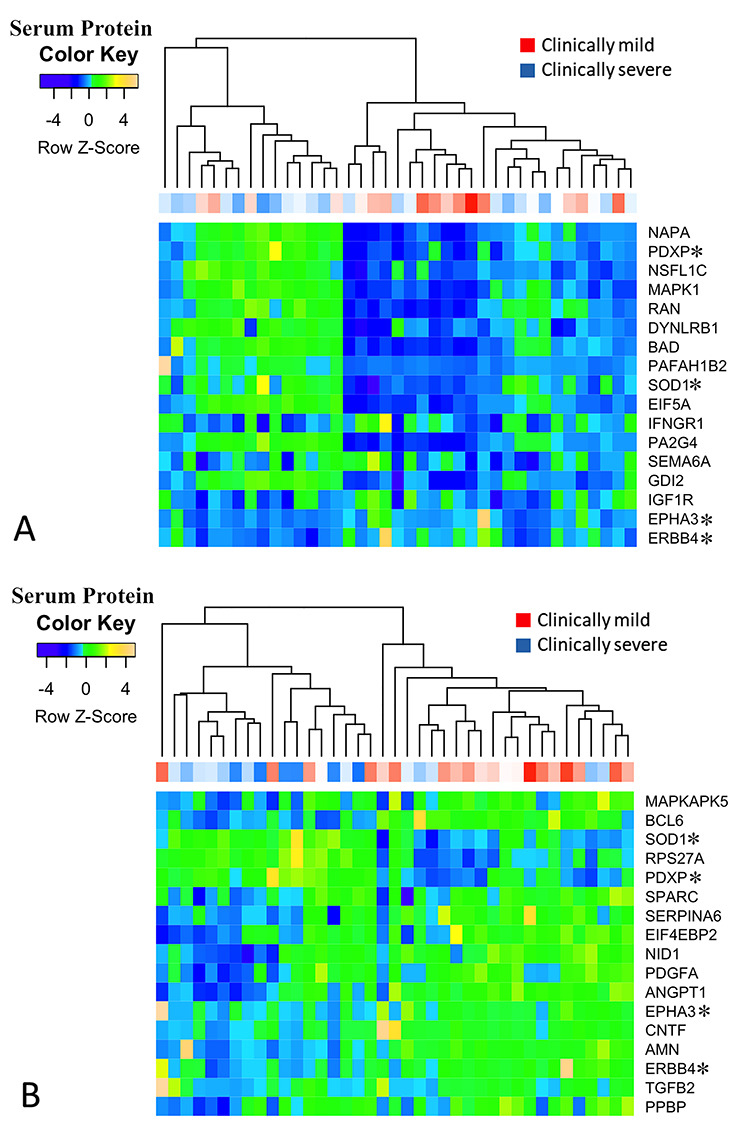
Clustering of serum proteins identified as associated with more affected phenotypes as determined by the outcome measures TTSTAND (**A**) and 6MWT (**B**) using unsupervised clustering. Patient-level clinical severity data is shown on the x-axis and serum protein levels on the *y*-axis. TTSTAND shows a clustering of clinically milder patients (red) in the center of dendrogram, corresponding to lower levels of most serum proteins from the model. 6MWT shows a clustering of clinically milder patients to the right of the dendrogram, corresponding to higher levels of most serum proteins in the model. Proteins marked with an asterisk are shared between the two clinical outcome models and demonstrate similar behavior in both models.

To visualize the performance of the selected serum proteins in differentiating between mildly and severely affected DMD patients at baseline, we converted the SOMAscan^®^ protein designations to more standard HGNC Approved Gene Symbols and generated heatmaps using unsupervised clustering (Tables 2 and 3; Fig. 4). For TTSTAND, data visualization showed that levels of most selected serum proteins were increased in patients with longer TTSTAND (more affected patients). For TTSTAND, three serum proteins showed clear reductions in more affected patients (IGF1R, EPHA3, ERBB4; see Table 2). For 6MWT, the opposite was found, where levels of most selected serum proteins showed decreased protein levels in patients with shorter 6MWT distances (more affected patients) (see Table 3). For 6MWT, three serum proteins showed increased levels in more affected patients (SOD1, RPS27A). The four serum proteins that were shared between the TTSTAND and 6MWT models behaved similarly in patient-level data (SOD1 and PDXP showed increased levels in severe patients; EPHA3 and ERBB4 showed decreased levels in severe patients).

**Table 2:** Serum proteins selected for TTSTAND regression model and individual performance in clinical and histological data sets

Target	UniProt	Entrez gene symbol	Elastic net selections (of 100 cross-validations)	Correlation serum protein versus clinical severity (*P*-value)	Biopsy mRNA normal versus DMD fold change (*P*-value)	Correlation mRNA versus histopathology severity score (*P*-value)
BAD	Q92934	BAD	100	−0.47 (0.013)	−1.2 (ns)	0.069 (ns)
EPHA3^*^	P29320	EPHA3	98	0.47 (0.014)	−1.1 (ns)	−0.033 (ns)
IGF-I sR	P08069	IGF1R	90	0.43 (0.020)	−1.1 (ns)	−0.24 (ns)
Semaphorin-6A	Q9H2E6	SEMA6A	90	−0.013 (ns)	−2.9 (2.9e−07)	−0.5 (0.023)
MK01	P28482	MAPK1	84	−0.53 (0.005)	−1.3 (0.007)	0.16 (ns)
eIF-5A-1	P63241	EIF5A	73	−0.3 (ns)	n/a	n/a
ERBB4^*^	Q15303	ERBB4	72	0.39 (0.034)	6.2 (9.5e−06)	0.66 (0.0037)
NSF1C	Q9UNZ2	NSFL1C	72	−0.31 (ns)	−1.4 (0.039)	−0.47 (0.031)
PAFAH beta subunit	P68402	PAFAH1B2	72	−0.33 (ns)	1.4 (0.039)	0.42 (ns)
SNAA	P54920	NAPA	72	−0.36 (0.049)	1.3 (0.002)	−0.57 (0.009)
OD^*^	P00441	SOD1	72	−0.38 (0.038)	1.2 (0.039)	0.58 (0.008)
DLRB1	Q9NP97	DYNLRB1	71	−0.54 (0.005)	1.1 (ns)	0.49 (0.024)
PLPP^*^	Q96GD0	PDXP	71	−0.26 (ns)	1.4 (0.039)	−0.28 (ns)
RAN	P62826	RAN	71	−0.36 (ns)	−1 (ns)	0.19 (ns)
PA2G4	Q9UQ80	PA2G4	70	−0.42 (0.021)	1.1 (ns)	0.03 (ns)
Rab GDP dissociation inhibitor beta	P50395	GDI2	69	−0.34 (ns)	−1.3 (0.003)	0.28 (ns)
IFN-g R1	P15260	IFNGR1	67	0.2 (ns)	−1.9 (0.0012)	−0.29 (ns)

List of proteins identified as explanatory variables from multivariate model for TTSTAND velocity along with repeatability of signal in multivariate model cross-validations, Spearman correlation of log-transformed mRNA values with severity (severe, moderate or mild histological severity in that order from DMD and BMD samples), fold change from mRNA (healthy controls, numerator versus DMD subjects, denominator) and Spearman correlation of log-transformed SOMAscan$^{\protect\textregistered} $ values with TTSTAND velocity. All *P*-values were adjusted using Benjamini–Hochberg (FDR) correction alongside the set of proteins for 6MWT distance with any *P*-value higher than 0.05 noted as ns. No ‘_at’ probeset was found for eIF-5A-1. Starred rows are proteins that are shared between the TTSTAND and 6MWT models.

**Table 3:** Serum proteins selected for 6MWT regression model and performance in correlations

Target	UniProt	Entrez gene symbol	Elastic net selections (of 100 cross-validations)	Correlation serum protein versus clinical severity (*P*-value)	Biopsy mRNA normal versus DMD fold change (*P*-value)	Correlation mRNA versus histopathology severity score (*P*-value)
Angiopoietin-1	Q15389	ANGPT1	98	0.53 (0.005)	−1.4 (ns)	0.15 (ns)
CBG	P08185	SERPINA6	98	0.44 (0.018)	1.8 (0.017)	0.35 (ns)
CTAP-III	P02775	PPBP	98	0.0044 (ns)	n/a	n/a
ERBB4^*^	Q15303	ERBB4	98	0.52 (0.005)	6.2 (9.5e−06)	0.66 (0.004)
TGF-b2	P61812	TGFB2	98	0.44 (0.018)	−1.7 (0.001)	0.018 (ns)
4EBP2	Q13542	EIF4EBP2	97	0.42 (0.021)	−1.1 (ns)	0.54 (0.013)
AMNLS	Q9BXJ7	AMN	97	0.22 (ns)	−2.3 (ns)	0.35 (ns)
EPHA3^*^	P29320	EPHA3	97	0.38 (0.04)	−1.1 (ns)	−0.033 (ns)
Nidogen	P14543	NID1	97	0.16 (ns)	−4.8 (5.2e−12)	−0.52 (0.016)
SOD^*^	P00441	SOD1	95	−0.32 (ns)	1.2 (0.039)	0.58 (0.008)
PDGF-AA	P04085	PDGFA	93	0.23 (ns)	1.2 (ns)	0.43 (ns)
ON	P09486	SPARC	74	0.28 (ns)	−3.6 (5.4e−14)	−0.54 (0.013)
BCL6	P41182	BCL6	71	0.31 (ns)	1 (ns)	0.39 (ns)
CNTF	P26441	CNTF	71	0.48 (0.013)	1.2 (0.039)	0.64 (0.004)
Ubiquitin+1	P62979	RPS27A	71	−0.35 (0.051)	−1.5 (9.3e−07)	−0.6 (0.007)
MAPK5	Q8IW41	MAPKAPK5	66	0.32 (ns)	1.4 (2.3e−05)	0.12 (ns)
PLPP^*^	Q96GD0	PDXP	61	−0.19 (ns)	1.4 (0.039)	−0.28 (ns)

List of proteins identified as explanatory variables from multivariate model for 6MWT distance along with repeatability of signal in multivariate model cross-validations, Spearman correlation of log-transformed mRNA values with severity (severe, moderate or mild histological severity in that order from DMD and BMD samples), fold change from mRNA (healthy controls, numerator versus DMD subjects, denominator) and Spearman correlation of log-transformed SOMAscan$^{\protect\textregistered} $ values with 6MWT distance. All *P*-values were adjusted using Benjamini–Hochberg (FDR) correction alongside the set of proteins for TTSTAND velocity with any *P*-value higher than 0.05 noted as ns. No ‘_at’ probeset was found for CTAP-III. Starred rows are proteins that are shared between the TTSTAND and 6MWT models.

### Validation using patient muscle biopsies

**Figure 5:**
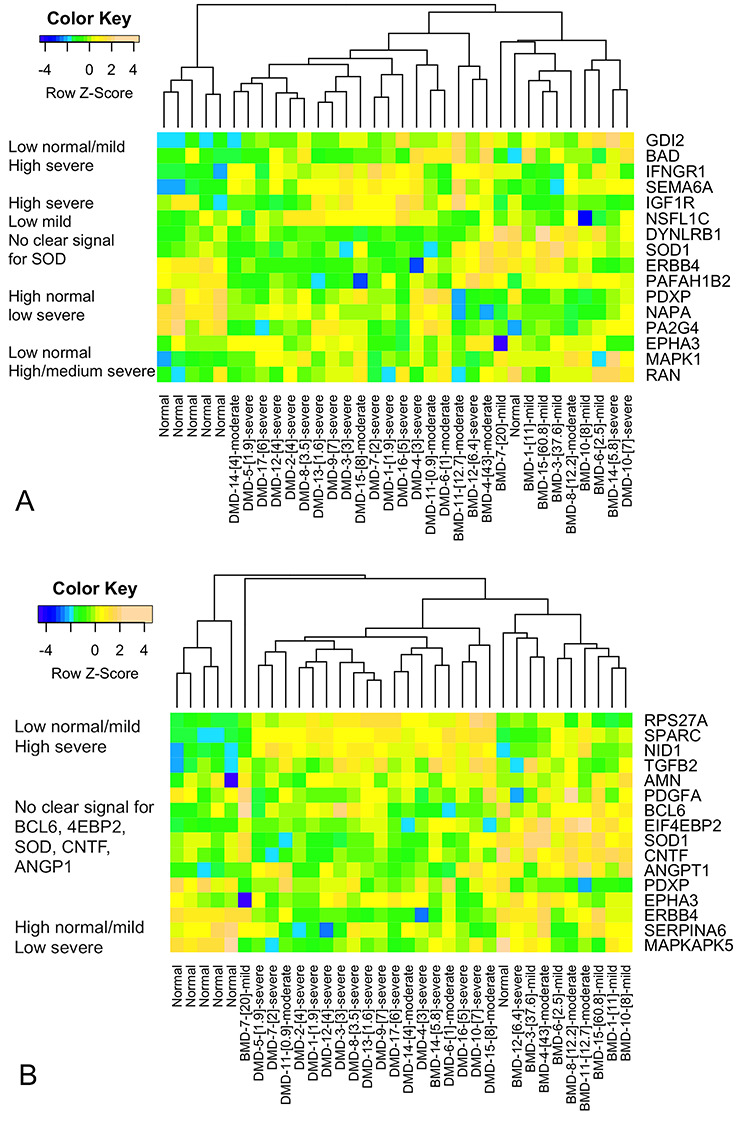
Unsupervised heatmap of muscle biopsies of defined histological severity clustered by mRNA expression levels of corresponding serum proteins from the TTSTAND (**A**) and 6MWT (**B**) models. mRNA levels were scaled, log-transformed data from a muscle biopsy data set on BMD, DMD and healthy control samples (2). The serum proteins selected by the TTSTAND and 6MWT models showed discriminatory power in differentiating muscle biopsies of variable histological severity, with distinct dendrogram branches for normal (left), mild (right) and severe (center) pathologies.

An independent sample set to validate the models was not available. A surrogate for clinical severity in dystrophinopathy (DMD/BMD) patients is muscle histological severity, where clinical outcomes correlate closely with the degree of fibrofatty replacement of muscle (fibrosis). Muscle histology as detected by MRI has been found to be the best biomarker of clinical findings in a series of Becker muscular dystrophy patients and to show superior predictive power than patient age, specific gene mutation or dystrophin protein levels in the muscle (21–23). Thus, we considered skeletal muscle biopsies from patients with defined histological severity as a surrogate biomarker for clinical severity. We have previously reported a patient muscle biopsy mRNA profiling data set with defined histological severity of dystrophinopathy patients (normal *n* = 6; dystrophinopathy *n* = 28) (NCBI GEO GSE109178) (2). Muscle histology was centrally read by a single reader and scored as normal, mild, moderate or severe histopathology. Unsupervised clustering was done using the mRNAs corresponding to the serum proteins selected by the baseline severity models for both TTSTAND and 6MWT (Fig. 5).

The majority of normal skeletal muscle biopsies clustered together to the left side of dendrograms, indicating differential expression of most selected serum proteins in DMD/BMD patient muscle. Also, for both TTSTAND and 6MWT, muscle samples with the most severe histology samples clustered in the center of the dendrogram, whereas most mild histology samples clustered to the right. This data suggests that the serum proteins selected by clinical outcome severity models were able to differentiate muscle biopsies of variable severity.

Each of the proteins identified as explanatory variables from multivariate model for TTSTAND and 6MWT clinical outcomes was tested for significance in the different data sets (Tables 1 and 2). Correlations of serum proteins with the outcome severity were tested using Spearman correlations (*n* = 39 patients), mRNA differential expression in normal (*n* = 6) versus DMD (*n* = 17) biopsies by *limma* package (24) for *R* and correlation of histological severity within dystrophinopathies with mRNA expression (*n* = 28). All *P*-values were adjusted using Benjamini–Hochberg false discovery rate (FDR) (25) correction (correction for TTSTAND velocity and 6MWT distance associated proteins conducted together but presented in their respective tables). No validated Affymetrix GeneChip probesets were found for eIF-5A-1 (TTSTAND) or CTAP-III (6MWT). Directionality of change was defined as ‘towards mild/normal’; if an increased serum protein showed association with a milder disease state or histology, this was indicated as a ‘+’ direction, and if increased in normal muscle versus DMD, this was also ‘+’. Note that for DMD versus normal muscle, this indication of directionality is the opposite of much of the disease literature, where a protein increased in DMD versus normal is indicated with a + sign (here we show this as a negative sign, as decreased in normal).

For TTSTAND, the three proteins with the most repeated signal in LASSO cross-validations showed moderate correlations with TTSTAND clinical outcome, with decreasing BAD serum levels (*r* = −0.47; *P* = 0.013) and increasing EPHA3 (*r* = +0.47; *P* = 0.014) and IGF-1 soluble receptor (IGF1R) (*r* = +0.43; *P* = 0.02) levels with milder clinical severity (Table 2). These same proteins showed no mRNA changes in DMD versus normal skeletal muscle, and mRNAs were not associated with histological severity. In contrast, serum semaphorin-6A protein levels showed no univariate correlation with TTSTAND clinical outcome (despite strong multivariate LASSO signal) but showed very strong upregulation of its mRNA in DMD muscle (−2.9-fold in normal muscle) and correlation with histological severity (*r* = −0.5; adjusted *P* = 0.023). ERBB4, a serum protein shared between TTSTAND and 6MWT models, showed strong and consistent associations across the three data sets, with increased serum levels associated with mild disease (univariate *r* = +0.39 and *r* = +0.52 for TTSTAND and 6MWT, respectively), significant upregulation of mRNA in normal muscle compared to DMD (+6.2-fold) and strong correlation with histological severity (*r* = 0.66). SOD1, another serum protein shared between TTSTAND and 6MWT models, showed significance in all three data sets, but the direction of change was opposite between serum protein (decreased expression in milder disease; *r* = −0.38) and mRNA in biopsy histological severity (increased expression in milder disease; *r* = +0.58). SNAA (NAPA) also performed well in all three data sets, with decreased serum protein (*r* = −0.36) and decreased muscle mRNA (*r* = −0.57) associated with milder disease.

For 6MWT, ERBB4 again performed well in both serum protein correlations (*r* = +0.52) and mRNAs in biopsies (same data as with TTSTAND) as did SOD1. Two biomarkers of muscle fibrosis, nidogen and ON/SPARC, showed moderate association with DMD versus normal mRNA (−4.8-fold and − 3.6-fold change in normal, respectively) and with histological severity (*r* = −0.52, *r* = −0.54), as is expected given that fibrosis is a major determinant of histological severity (indeed, these mRNAs serve as a positive control for biopsies). Note that these same two proteins, while selected by multivariate linear models, showed poor univariate correlation with 6MWT clinical outcome. Ubiquitin+1 (RPS27A) behaved similarly to nidogen and ON/SPARC mRNAs in the muscle, and this is likely a novel biomarker of muscle fibrosis. CNTF was also moderately associated with clinical and histological outcomes, with increased serum protein associated with milder disease (*r* = +0.48) and mRNA with milder histological severity (*r* = +0.64).

Data for all data sets were summarized in terms of directionality and significance in serum and muscle, with comments on protein function in Supplementary Material, Table S3. Potential or known biological significance of these findings is provided in the Discussion.

### Testing of models in additional clinical outcomes

An alternative approach to validation of the regression models for serum biomarkers is to test these models in additional clinical outcomes collected in the VBP15–002/VBP15–003 clinical trial. Given the shared proximal strength latent factors behind TTSTAND, TTCLIMB and TTRW velocities, the 17 TTSTAND velocity-associated identified proteins were used to model velocity measures for TTRW (time to run/walk 10 m) and TTCLIMB (time to climb four stairs). A ridge regression using all 17 proteins yielded a squared correlation of 0.76 between observed and predicted TTCLIMB velocity. Similarly, a ridge regression using all 17 proteins yielded a squared correlation of 0.50 between observed and predicted outcomes for TTRW velocity.

The coefficients obtained from these two models were concordant (same directionality) for 15 out of 17 biomarkers for the TTSTAND, TTCLIMB and TTRW models; the estimated coefficient for PAFAH1B2 is in the opposite direction for TTRW velocity as compared to the TTSTAND and TTCLIMB velocity models. Similarly, the coefficient for IFNGR1 is estimated in the opposite direction for the TTCLIMB velocity model as compared to the other two.

### Mapping of selected proteins to biochemical networks

Ingenuity pathway analysis (IPA) was used to map the selected proteins to biochemical/molecular networks supported by the literature (Supplementary Material, Fig. S1 [TTSTAND]; Supplementary Material, Fig. S2 [6MWT]). The correlation of mRNA change with histological severity was used to indicate direction of change in (high in more severe = red color; low in more severe = green); this is the opposite orientation as in Tables 1 and 2 but is more consistent with the literature for pathological tissues.

For TTSTAND, the top-ranked protein network connecting proteins in the model contained IGF1R, ERBB4, EPHA3, BAD, SEMA6A and SOD1 and involved cell growth and signaling networks (Supplementary Material, Fig. S1). These pathways are most closely related to muscle regeneration and consistent with muscle strength phenotypes.

For 6MWT, the top-ranked networks contained extracellular matrix proteins (NID1, PDGFA, CNTF, SPARC and ANGPT1), plasma membrane proteins (EPHA3, ERBB4) and cytosolic (MAPKAP5, EIF 4EPB2, BCL6, SOD1) (Supplementary Material, Fig. S2). This is consistent with progressive fibrosis pathways that are strongly correlated with muscle function.

## Discussion

This study sought to identify a set of serum proteins that were correlated with clinical severity of DMD within a narrow age window (3 years), at an early stage of the disease (4 to <7 years), and participants were treatment-naïve (steroid-naïve). The study was carried out in a clinical trial setting, with robust standard operating procedures for serum sample collection and storage. We used the SOMAscan^®^ proteomics platform to carry out a broad assessment of serum proteins. The strengths and weaknesses of the SOMAscan^®^ platform have been reviewed in detail (26), and this platform has been used for multiple previous studies of serum from DMD (10–12,27). Briefly, the strength of the aptamer-based platform is that it tests three dilutions of sera over a 10 000-fold concentration range and is highly parallel (1305 serum proteins tested). Weaknesses of the platform include influence of genetic polymorphisms on aptamer binding to encoded proteins and concerns regarding specificity of aptamers to some target proteins (28).

Other recent reports have studied serum proteins diagnostic of DMD versus controls, and proteins correlated with age and disease progression (10,11,27,29). Additional reports have looked at muscle biopsies from DMD patients and defined proteins and mRNAs associated with disease progression (30,31). We checked for overlap with protein lists in recent reports (29,31) and found overlap regarding SOD and NSFL1C in a report providing muscle biopsy changes in DMD versus normal versus Becker muscular dystrophy (31). SOD1 was downregulated in DMD muscle, while NSFL1C was upregulated in the biopsies, consistent with our findings. Our approach differed substantially from previous reports in that instead of studying age-related and drug-related responses of biomarkers, we removed these variables and focused only on disease severity in young, drug-free patients. To the authors’ knowledge, this is the first effort at defining biological correlates of early-age severity using a multivariate biomarker-based model in DMD, unencumbered by confounding due to wide age ranges and previous corticosteroid exposure. Interestingly, creatine kinase (CK) was tested on the SOMAscan^®^ panel, but not selected as associated with clinical severity in our study (univariate Spearman correlation of CKM, UniProt = P06732, with TTSTAND velocity = −0.12 and 6MWT distance = −0.1). Serum CK is known to be highly variable even with repeated measures within a DMD subject and also known to decrease markedly with age.

Rather than identify single serum proteins correlated with clinical severity, the goal of this study was to identify biomarkers that, in combination, could model early-age severity and so, all proteins that may be associated with varying levels of severity should be considered. Accordingly, all 1305 quantified proteins were used, and no pre-filtering by differential expression of DMD subjects versus healthy controls was done. Instead, we relied on weighted correlation network analysis to cluster the proteins into homogenous groups and only considered the groups that were associated with specific outcomes. This technique has previously been demonstrated to be more often more powerful than using an approach based on standard marginal correlation-based screening which may ignore correlations between protein profiles. The network approach does better by using ‘connectivity’ or ‘adjacency’ which lead to more biological significance, i.e. signal rather than noise. This also helps with power concerns. Clusters of proteins that are significantly associated with DMD-relevant outcomes were identified. Highly connected proteins from modules significantly correlated with clinical outcome measures were considered as potentials for modeling DMD severity. The use of such an approach ensures that the subset of biomarkers identified as candidate biomarkers are less likely to have spurious correlations to outcomes.

We report analysis of highly standardized clinical phenotyping data from 39 DMD steroid-naïve boys in a narrow age range (4 to <7 years) to generate models of sets of serum proteins associated with phenotypic differences. While all patients with DMD share the same molecular and biochemical feature of loss of the dystrophin protein in muscle (1,32), the onset and progression of the disease varies from patient to patient. Our goal was to gain insights into the molecular mechanisms of variable disease severity in this monogenic disorder by correlating serum proteomic profiles (1305 proteins assessed using SOMAscan^®^) with specific clinical outcomes. We report the reliability of the five clinical outcomes studied in this current report (time to stand from the floor [TTSTAND], time to run/walk 10 m [TTRUN], time to climb 4 stairs [TTCLIMB], 6-minute walk test meters walked [6MWT], and 17-test NSAA) in Table 1. Each of these clinical outcomes is routinely utilized in clinical trials, and in this data showed %CV of repeated measures ranging from 7.17% (6MWT) to 16.17% (TTSTAND velocity).

Two clinical outcomes were studied for biomarker associations: TTSTAND velocity (muscle strength) and 6MWT (endurance). Each outcome was measured on four separate occasions in each patient over a 6-week time frame, with means of each measure then used to build models. Models were built using a multistage data reduction approach (WGCNA), followed by multivariate linear modeling and validations (Elastic Net and LASSO) (Fig. 1). Separate models were built for TTSTAND and 6MWT, and the selected proteins (17 serum proteins in each model) then tested in patient muscle biopsies of different histological severity, as well as testing in the additional outcomes (TTRW and TTCLIMB velocities).

Of the muscle function tests typically performed by patients with DMD in clinical research, TTSTAND is the most physically demanding because it requires proximal, lower extremity muscle strength that is lost relatively early in the disease process. TTSTAND values at a young age have been found to be highly predictive of functional loss at later ages (3). It is the primary outcome in the vamorolone clinical trials for measuring drug efficacy (13). Seventeen proteins were selected in the final LASSO model, with elastic net initially selecting these model components in 67–100 cross-validations (Table 2). We tested the 17 proteins in mRNA profiling data sets we have previously reported from normal skeletal muscle biopsies (*n* = 6) and dystrophinopathy patient biopsies of variable histological severity (*n* = 28) (NCBI GEO GSE109178) (2). Key findings of these modeling studies were as follows. (1) About half of proteins were individually correlated with TTSTAND clinical outcomes, whereas the other half only performed well within the multivariate linear model. (2) The proteins in the model generally were involved in cell growth and tissue repair (Supplementary Material, Fig. S1). A subset showed strong correlation with both clinical severity and histological severity (ERBB4, SNAA, SOD1, DLRB1). Two serum biomarkers showed change in opposite directions in serum and skeletal muscle (SOD1, DLRB1). SOD1 (superoxide dismutase) is a well-characterized protein involved in protection against oxidative stress. In skeletal muscle, SOD1 mRNA showed increased expression in histologically less affected muscle (*R* + 0.58; adjusted *P* = 0.008) but decreased as a serum protein in milder clinical disease (*R* − 0.38; adjusted *P* = 0.038). DLRB1 (DYNLRB1) behaved very similarly to SOD1 (Table 2). DLRB1 has been found to be important in TGF-β signaling (33), and TGF-β is central in the progressive pathophysiology of DMD (2,30). Both SOD1 and DLRB1 show relatively high level of expression in most tissues and cells. Thus, the serum origin of these two proteins may not be from skeletal muscle, and this may explain the discordant findings between DMD blood and muscle.

Meters walked in the 6MWT has been used as the primary outcome measure in a number of DMD drug development programs. As a secondary outcome measure in the vamorolone clinical trials, 6MWT showed a broad dynamic range and strong dose-dependent improvements in response to vamorolone treatment (13). 6MWT is thought to be more reflective of muscle endurance, whereas TTSTAND is more reflective of proximal muscle strength. Systematic dimension reduction and regression modeling selected 17 serum proteins associated with clinical severity measured by 6MWT distance (Table 3). These proteins clearly centered on muscle fibrosis pathways (Supplementary Material, Fig. S2), with osteonectin/SPARC, TGFB2, ubiquitin (RPS27A) and PDGR associated with development of fibrosis in DMD (34–37).

Four serum proteins were selected in both TTSTAND and 6MWT models: EPHA3, ERBB4, SOD1 and PLPP/PDXP (Tables 1 and 2). It is not surprising that proteins were shared as overlapping modules were fed into model building (Fig. 1). SOD1 was discussed above and behaved similarly for both clinical outcomes. ERBB4 showed disease relationships for all data sets, with high serum levels associated with milder disease for both TTSTAND (*r* = +0.39), 6MWT (*r* = +0.52) and muscle histology (*r* = +0.66). Further, ERBB4 mRNA was strongly downregulated in DMD muscle (−6.2-fold; adjusted *P* = 9.5 × 10^−6^). ERBB4 is a cell-surface receptor neuregulin, and the NRG1/ERBB4 pathway has been extensively studied in hypoxia, TGF-β and fibrosis in the heart, kidney and neuronal cells where it plays a protective role and can be downregulated in disease states (38,39). In skeletal muscle, it appears important for mitochondrial function, and deficiency can lead to defects in oxidative phosphorylation (40). Mitochondrial dysfunction and insufficient oxidative metabolic capacity is increasingly recognized as a key part of DMD pathophysiology (41,42). It is tempting to speculate that serum ERBB4 levels could be a biomarker for mitochondrial dysfunction in skeletal muscle in DMD. This is further supported by a recent study showing that decreased levels of circulating of ERBB4 ectodomain is strongly associated with impaired ERBB4 pathway in cerebrospinal fluid and plasma of ALS patients (43). ERBB4 is also highly expressed in smooth muscle as is dystrophin, while smooth muscle lacks dystrophin in DMD. Thus, ERBB4 in DMD sera may reflect release of this protein from smooth muscle into the bloodstream and a vascular effect on the dystrophic phenotype.

Note that some proteins are only selected as important in the multivariate models (e.g. semaphorin-6A [TTSTAND] and CTAP-III [6MWT]) which are not individually correlated to clinical outcomes but was correlated to an eigenprotein. In a multivariate model, such proteins are being selected because of correlation with outcome after adjusting for other proteins already in the model. While this is a relatively large sample size for a DMD study, typically such biomarker-related model investigations have been conducted with larger number of subjects stratified into test and validation groups (44). While we utilized 10-fold (and repeated) cross-validation in model building, our protein clusters as a first stage of dimension reduction were formed based on all 39 samples. Hence, overfitting is a possibility, and validation on an external protein data set is required. We relied on biological validation and visually test if these biomarkers are differentially expressed in DMD muscle, associated with patient diagnosis and with the extent of pathology.

Bioinformatic approaches have previously been used to define phenotype-associated signatures. For example, histological data and omics on human muscle biopsies from molecularly defined disorders were integrated to better understand pathophysiological processes in muscle disease (45,46). Clusters of transcripts with a 56-member TGFβ-centered network consistent with tissue fibrosis were identified (2). X-ROS was identified as amplifying Ca^2+^ influx in adult mdx mice with transcriptome analysis identifying increased expression of X-ROS-related genes in human DMD skeletal muscle (47). Statistical models have being increasingly used with biomarkers to predict onset (48), model risk prediction (44), identify highly associated biomarkers from cancer exosomes (49) and identify co-regulated groups of circulating proteins demonstrating close relationships to disease states (50).

Here, we constructed robust biomarker-based multivariate models for early-age severity using TTSTAND and 6MWT data as proxies for severity/muscle function. This age range is especially of interest because of the potential for therapeutic interventions. These models may help towards monitoring clinical response using biomarkers and allow for personalized predictions of response in trials and in clinical practice.

Considering these biomarkers as prognostic or predictive will require a specific context of use and clinical validation. A limitation of our study is that we have not demonstrated longitudinal sensitivity to age or drug–response; it needs further study. Other limitations are the lack of an independent clinical validation set (independent cohort) and the need to validate the SOMAscan^®^ assay findings by another method of detection.

The results reported here suggest that muscle remodeling, and growth pathways may be more correlated with TTSTAND (a measure of proximal strength), whereas fibrosis pathways are more correlated with 6MWT (a measure of endurance). These signatures can be detected in blood and should be further studied as possible biomarkers of disease progression and/or drug response.

## Materials and Methods

### Patients, phenotyping and serum proteins

The vamorolone VBP15–002 study enrolled 48 steroid-naïve DMD boys between 4 and 7 years of age in a multicenter CINRG clinical trial (12). The design and results of the trial, including inclusion and exclusion criteria, ethics review and data collection, have been published. The program carried out broad serum biomarker studies, with 1305 proteins tested (SOMAscan^®^ aptamer arrays) on each patient. In this current study, data from serum samples taken at baseline (prior to any drug treatment) were studied for associations with clinical outcome measures at baseline.

Prior to conducting the VBP15–002 clinical trial, a set of serum biomarkers, assayed by SOMAscan^®^ aptamer arrays, had been defined as responsive to corticosteroid treatment in four disease states (DMD, inflammatory bowel disease, ANKA-associated vasculitis [AAV], juvenile dermatomyositis) (14,51). Based on this data, in the initial analyses of the DMD patients enrolled in VBP15-002, 13 proteins were pre-specified as either exploratory biomarkers for either safety (*n* = 6 proteins) or efficacy (*n* = 7 proteins) from the 1305 assayed (12). A data mask was applied to the SOMAscan^®^ aptamer profiles to study only these 13 proteins in the previous report. Here, we removed the data mask and considered all 1305 proteins in the current analysis.

The data set studied in this current analysis was the de-identified data on all 1305 serum biomarkers in blood samples taken at baseline and 5 clinical outcome measures (6 min walk distance [6MWT; in meters], time to rise from supine [TTSTAND; in velocity rise/s], time to climb 4 stairs [TTCLIMB; in velocity rise/s], time to run/walk 10 m (TTRW; in velocity m/s] and NSAA (score). The NSAA is a composite score of 17 clinical tests and abilities, where each test is scored as 0 (unable to do test), 1 (ability to do test but not normal) to 2 (normal ability to do test), with the maximum possible score of 17 × 2 or 34 points.

Children that are only 4 to <7 years of age can show variable reliability in outcomes, due to challenges in following commands, variability in mood and other confounding variables beyond motor function capability. The reliability (quantified as percent coefficient of variance; %CV) for each of the five outcome measures was studied by testing each child in the clinical trial four times over a period of about 6 weeks. For the purposes of this current study, the average of the four repeated clinical outcome measurements (screening, baseline, 2-week treatment, 2 weeks off treatment) over the 6 weeks was utilized as outcomes for our models. Note that while two of those outcome measurements were taken after the starting and stopping of vamorolone therapy, vamorolone does not have any significant effect on clinical outcomes in 2 weeks (12). Some subjects had less than four measurements; all available measurements were used, except for two subjects where rescreening measurements were used in lieu of the screening measurement after re-vaccination for varicella. For the timed function tests, we use the reciprocal of the times to convert to velocity units (for TTRW, 10 m is divided by the observed times), as is standard practice; longitudinally, this has the advantage of resulting in a linear pattern of decline (52).

The biomarker measurements are high-quality proteomic profiling data (SOMAscan^®^ Assay; 1305 biomarkers) at baseline for 39 subjects. The raw data generated from the SOMAscan^®^ assay was hybridization control normalized and median signal normalized.

We checked for clear outliers in biomarker values and outcome measurements based on distance from the mean. An unsupervised hierarchical clustering based on protein values also did not show any outliers. Principal component analysis of outcome variables and multidimensional scaling analysis of proteins were conducted as part of exploratory analysis.

### Weighted correlation network approach

For all data analysis, the R statistical computing software (53) was used. As we had 1305 biomarkers but only 39 subjects, dimension reduction that preserved relationships between the proteins was important. Because correlated proteins are likely to be functionally associated and involved in functional pathways, we define modules (aka clusters) of correlated proteins. A protein similarity network was constructed using a weighted correlation network approach (15,18). A hierarchical clustering of proteins based on a transformation of pairwise correlations is used to identify clusters. This is conducted in a completely unsupervised manner (not connected to any outcomes or traits) at this stage. The first step of filtering 1305 biomarkers involves unsupervised clustering of the 1305 biomarker expression values. Because of the way SOMAscan^®^ data is generated using three different dilutions of input sera over a >10 000-fold dynamic range, we are only using correlations, i.e. we rely on relative trends here, not the obtained relative frequency units.

A correlation-based network was built in two parts. First, a co-expression network was built based on correlations between pairwise biomarkers raised to a power chosen based on a scale-free topology criterion (using this, the differences between a high and low correlation are accentuated). We used biweight midcorrelations, which are a robust alternative to Pearson correlations for dealing with any possible extreme values. Then, a topological overlap matrix was constructed, which builds an adjacency measure based on topological similarity, i.e. it considers two proteins as proximal by inspecting shared neighbors and using connection strength to other proteins. This makes the network less sensitive to spurious connections or to connections missing due to random noise (54). Clusters were obtained using this topological overlap measure as a distance measure in an unsupervised (proteins only, no traits) hierarchical cluster analysis. All of the statistical procedures carried out are purely part of screening and completely unsupervised.

#### Constructing models

We computed a summary measure of each module, a ME of a given cluster (module). This is basically the first principal component for this module and is a weighted sum of the expression values of the proteins assigned to this cluster. This captured the highest amount of variation that a principal component can model for this cluster. Capturing the variation is important. We obtained correlations of clinical outcomes with the MEs to define important correlated MEs, and all proteins correlated with these important MEs (corresponding *P* value less than 0.15; chosen for screening for higher power because of small sample size) are carried forward. This means that we are carrying forward only proteins that are associated with DMD-relevant clinical outcomes.

The above list of candidate proteins was further refined by two measures, the first is how similar individual proteins are to the MEs and the second is how connected they are to other proteins within their modules, using biweight midcorrelations. These were selected based on specific cutoffs (discussed below).

We focused discovery on two clinical outcomes: TTSTAND (strength) and 6MWT (endurance). We chose a subset of between 10 and 20 proteins that were associated with these two clinical outcomes. To drop the proteins that are not helpful for explaining observed variation, we used regularization techniques from the *glmnet* package (55) for *R* to construct linear models. LASSO and elastic net are variable selection techniques that allow for fitting linear models with more covariates than samples. An elastic net can better deal with correlations in explanatory variables while LASSO is better for variable selection. Here, we used elastic net (mixing parameter α = 0.95 to make it behave more like a LASSO) to get the best of both worlds (55), choosing the penalty parameter as the one that results in minimum mean cross-validated error.

Model development can be dependent on the training/validation data split, and some proteins may be picked out more often than others based on the split; therefore, we did 100 10-fold cross-validations (repeated random-split cross-validation) (44) to identify the set of biomarkers that elastic net selects at least 60 times out of the 100 cross-validations. We did this for a range of cutoffs (correlations between 0.5 and 0.75 for similarity to ME and standardized connectivity within cluster) and chose a large cutoff (yielding highly connected candidate proteins that drive the variation in the cluster) that resulted in a relatively large set (10 to 20) of proteins. For time to stand velocity, we used 0.70 for the cutoffs, while for 6-min walk-test, we used 0.63. This was followed up with one final LASSO model to confirm whether these proteins would be retained in the presence of the other identified proteins. This process enabled us to identify two sets of biomarkers, one for early-age severity as quantified through time to stand velocity (proximal strength) and another for 6-min walk test (endurance). There were some possible outliers in the identified proteins and during model development; we did not use any transformation on them or remove the outliers; further, we calculated correlation between predicted and observed outcomes after removing these possible outliers, and there was very little difference compared to using all the observations.

#### Measures of mRNA in patient biopsies corresponding to selected serum proteins

To determine if serum proteins correlated with clinical severity showed gene expression in patient muscle and if expression changed as a function of disease state of muscle, we accessed publicly available mRNA profiles we had previously generated from 49 human patient mRNA profiles generated using HG-U133 Plus 2.0 microarrays (2). We limited queries to 34 profiles (controls and dystrophinopathies) (*n* = 6 normal controls, *n* = 17 DMD and *n* = 11 Becker muscular dystrophy). Note that as compared to our model-building data set, subjects had a wide range of ages, severity of their condition and histopathological findings. Affymetrix IDs were converted to UniProt accessions using bioDBnet (56). Only probes designed to detect the antisense strand of the gene of interest (annotated with ‘_at’) were used here. However, some UniProt accessions still had multiple probe sets (which may detect alternative isoforms of the mRNA). We used those probes that had maximum variance (to capture intergroup variation as well) compared to the other probes for a specific UniProt accession. We did an unsupervised clustering (agnostic to diagnosis) of the candidate efficacy biomarkers to visually test if these biomarkers are differentially expressed in DMD muscle, associated with patient diagnosis, and if they are associated with the extent of pathology.

## Supplementary Material

Dang_et_al_Supplementary_Material_1_ddaa132
