# Supplementary material for: Serum biomarkers associated with baseline clinical severity in young steroid-naïve Duchenne muscular dystrophy boys
Source: Hum Mol Genet. 2020 Jun 27;29(15):2481–95. doi: 10.1093/hmg/ddaa132 (PMC7471506; doi:10.1093/hmg/ddaa132)
Supplement: Dang_et_al_Supplementary_Material_1_ddaa132 [file dang_et_al_supplementary_material_1_ddaa132.docx]

**Supplementary Material for**

**Serum biomarkers associated with baseline clinical severity in young steroid-naive Duchenne muscular dystrophy boys**

Utkarsh J. Dang^1*^, Michael Ziemba^2^, Paula R. Clemens^3^, Yetrib Hathout^4^, Laurie S. Conklin^5^, CINRG Vamorolone 002/003 Investigators, and Eric P. Hoffman^4,5^

| Name | Uniprot ID | Estimate |
| --- | --- | --- |
| (Intercept) |  | 1.1 |
| BAD | Q92934 | -0.12 |
| EPHA3 | P29320 | 0.058 |
| IGF-I sR | P08069 | 0.24 |
| Semaphorin-6A | Q9H2E6 | -0.27 |
| MK01 | P28482 | -0.13 |
| eIF-5A-1 | P63241 | 0.1 |
| ERBB4 | Q15303 | 0.031 |
| NSF1C | Q9UNZ2 | 0.042 |
| PAFAH beta subunit | P68402 | -0.0093 |
| SNAA | P54920 | 0.24 |
| SOD | P00441 | -0.12 |
| DLRB1 | Q9NP97 | -0.0021 |
| PLPP | Q96GD0 | 0.017 |
| RAN | P62826 | -0.017 |
| PA2G4 | Q9UQ80 | -0.044 |
| Rab GDP dissociation inhibitor beta | P50395 | -0.12 |
| IFN-g R1 | P15260 | 0.076 |

**Supplementary Table 1: Names and Uniprot IDs of selected serum proteins quantified using SOMAscan® along with the LASSO estimates for the time to stand velocity model**

| Name | Uniprot ID | Estimate |
| --- | --- | --- |
| (Intercept) |  | -190 |
| Angiopoietin-1 | Q15389 | 230 |
| CBG | P08185 | 200 |
| CTAP-III | P02775 | -150 |
| ERBB4 | Q15303 | 70 |
| TGF-b2 | P61812 | 67 |
| 4EBP2 | Q13542 | 66 |
| AMNLS | Q9BXJ7 | -35 |
| EPHA3 | P29320 | 27 |
| Nidogen | P14543 | -66 |
| SOD | P00441 | -64 |
| PDGF-AA | P04085 | -74 |
| ON | P09486 | 24 |
| BCL6 | P41182 | -100 |
| CNTF | P26441 | 8.6 |
| Ubiquitin+1 | P62979 | -31 |
| MAPK5 | Q8IW41 | 31 |
| PLPP | Q96GD0 | 18 |

**Supplementary Table 2: Names and Uniprot IDs of selected serum proteins quantified using SOMAscan® along with the LASSO estimates for the six-minute walk test model.**

|  | **Gene Symbol** | **Elastic net signal repeatability** | **Increased serum levels associated with** | **Expression in DMD muscle vs. normal** | **Increased muscle expression associated with** | **Biological role** |
| --- | --- | --- | --- | --- | --- | --- |
| **TTSTAND** strength | | | | | | |
| BAD | BAD | 100/100 | *Severe disease* | - | - | Agonist of cell death; mitochondrial function |
| EPHA3 | EPHA3 | 98/100 | **Milder disease** | - | - | Neuronal guidance |
| IGF-1 sR (soluble receptor) | IGF1R | 90/100 | **Milder disease** | - | *-* | Growth |
| Semaphorin-6A | SEMA6A | 90/100 | *Severe disease* | High | *Severe histopath* | Neuronal guidance |
| MK01 | MAPK1 | 84/100 | *Severe disease* | High | - | Signal transduction; TGFb |
| eIF-5A-1 | EIF5A | 73/100 | *Severe disease* | n/a | n/a | Protein translation |
| ERBB4 | ERBB4 | 72/100 | **Milder disease** | Low | **Mild histopath** | Cleavable signal transduction |
| NSF1C | NSFL1C | 72/100 | *Severe disease* | High | *Severe histopath* | Membrane fusion |
| PAFAH beta subunit | PAFAH1B2 | 72/100 | *Severe disease* | Low | **-** | Platelet activation |
| SNAA | NAPA | 72/100 | *Severe disease* | Low | *Severe histopath* | Vesicle fusion |
| SOD | SOD1 | 72/100 | *Severe disease* | -Low | **Mild histopath** | Anti-oxidant |
| DLRB1 | DYNLRB1 | 71/100 | *Severe disease* | - | **Mild histopath** | Dynein family |
| PLPP | PDXP | 71/100 | *Severe disease* | Low | *-* | Actin reorganization |
| RAN | RAN | 71/100 | *Severe disease* | - | - | Microtubule organization |
| PA2G4 | PA2G4 | 70/100 | *Severe disease* | Low | - | Cell survival |
| Rab GDP dissociative inhibitor beta | GDI2 | 69/100 | *Severe disease* | High | - | Vesical transport |
| IFN-g R1 | IFNGR1 | 67/100 | *Severe disease* | High | *-* | Anti-fibrogenic; upregulates IL12, CD64 |
| **6MWT** endurance | | | | | | |
| Angiopoietin-1 | ANGPT1 | 98/100 | **Milder disease** | - | **-** | Angiogenic, anti-inflammatory |
| CBG | SERPINA6 | 98/100 | **Milder disease** | Low | **-** | Cortisol-binding; stabilization |
| CTAP-III | PPBP | 98/100 | **Milder disease** | n/a | n/a | Platelet associated; connective tissue activating |
| ERBB4 | ERBB4 | 98/100 | **Milder disease** | Low | **Mild histopath** | Cleavable signal transduction |
| TGF-b2 | TGFB2 | 98/100 | **Milder disease** | High | *-* | Fibrosis in DMD |
| 4EBP2 | EIF4EBP2 | 97/100 | **Milder disease** | - | **Mild histopath** | Translational control |
| AMNLS | AMN | 97/100 | **Milder disease** | - | - | Kidney and gut epithelial |
| EPHA3 | EPHA3 | 97/100 | **Milder disease** | - | - | Neuronal guidance |
| Nidogen | NID1 | 97/100 | **Milder disease** | High | *Severe histopath* | Basement membrane; links ColIV and Laminin |
| SOD | SOD1 | 95/100 | *Severe disease* | Low | **Mild histopath** | Anti-oxidant |
| PDGF-AA | PDGFA | 93/100 | **Milder disease** | - | - | Platelet-derived; fibrosis |
| ON | SPARC | 74/100 | **Milder disease** | High | *Severe histopath* | Endothelial; response to injury |
| BCL6 | BCL6 | 71/100 | **Milder disease** | - | **-** | Mitosis in B cells |
| CNTF | CNTF | 71/100 | **Milder disease** | Low | **Mild histopath** | Neurotophic |
| Ubiquitin+1 | RPS27A | 71/100 | *Severe disease* | High | *Severe histopath* | Protein degradation |
| MAPK5 | MAPKAPK5 | 66/100 | **Milder disease** | Low | - | Activated in cell stress |
| PLPP | PDXP | 61/100 | *Severe disease* | Low | - | Actin reorganization |

**Supplementary Table 3: Names and gene symbols of selected proteins along with directionality, significance in serum and muscle, and comments on protein function. For serum levels, directionality in multivariate model is noted whereas for mRNA levels, annotation is provided if the false discovery rate-corrected p-value was significant.**


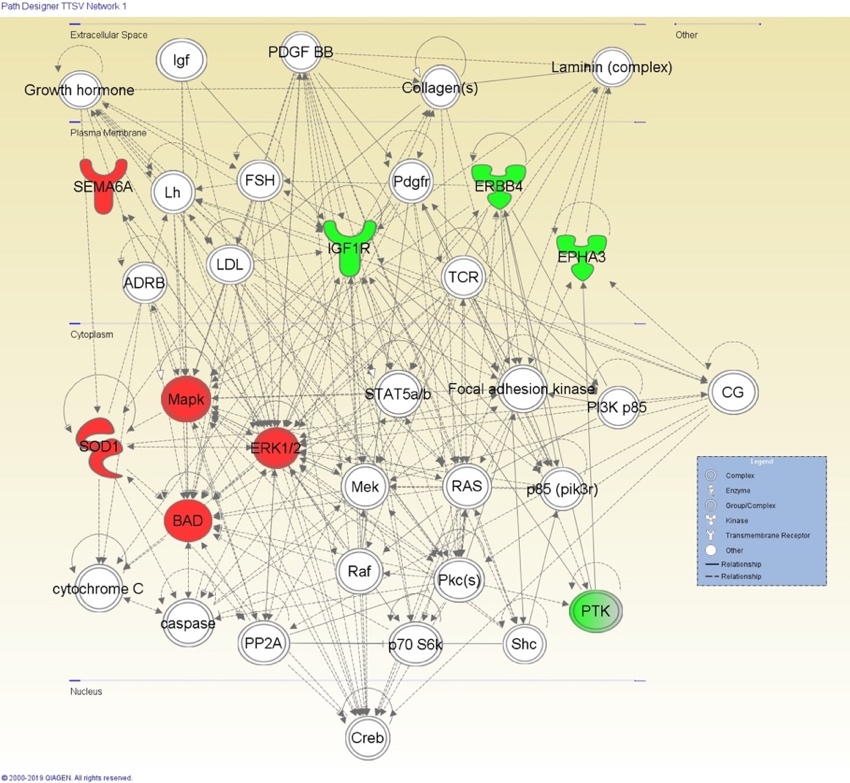


**Supplemental Figure 1: Significant networks produced by Ingenuity Pathway Analysis for proteins identified for time to stand velocity. Red symbols are serum proteins and/or muscle biopsy mRNAs that are generally increased in more severe patients, whereas green symbols are those that are decreased in more severe patients. The IPA network score assigned to this top-ranked network was 18.**


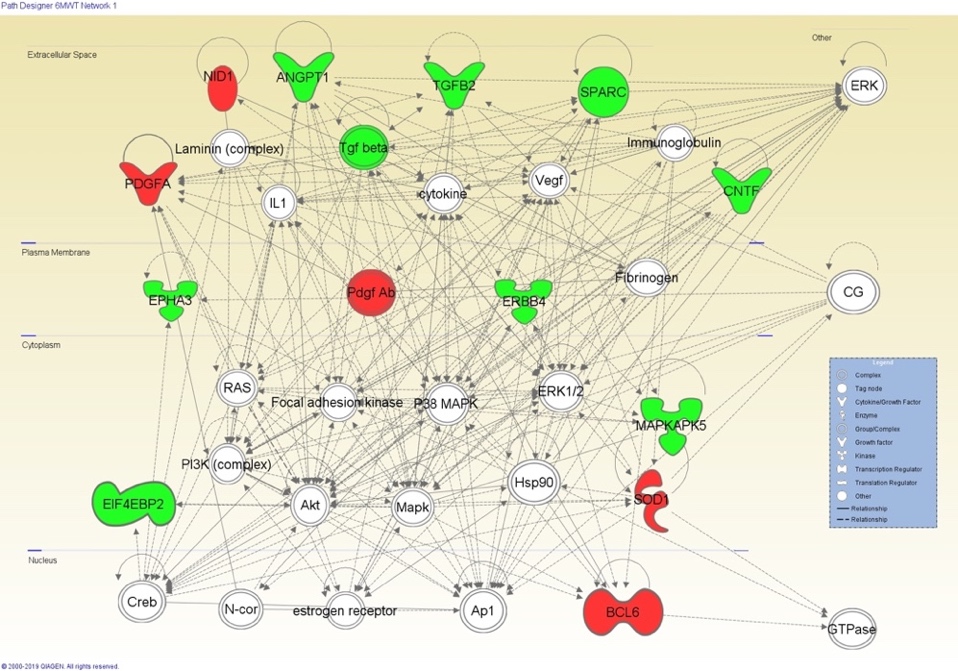


**Supplemental Figure 2: Significant networks produced by Ingenuity Pathway Analysis for proteins identified for 6MWT. Red symbols are serum proteins and/or muscle biopsy mRNAs that are generally increased in more severe patients, whereas green symbols are those that are decreased in more severe patients. The IPA network score assigned to this top-ranked network was 21.**
